# A structural basis for lithium and substrate binding of an inositide phosphatase

**DOI:** 10.1074/jbc.RA120.014057

**Published:** 2020-11-24

**Authors:** D. Eric Dollins, Jian-Ping Xiong, Stuart Endo-Streeter, David E. Anderson, Vinay S. Bansal, Jay W. Ponder, Yi Ren, John D. York

**Affiliations:** 1 Department of Pharmacology and Cancer Biology, Duke University, Durham, North Carolina, USA; 2 Department of Biochemistry, Vanderbilt University, Nashville, Tennessee, USA; 3 Department of Chemistry, Washington University, St Louis, Missouri, USA

**Keywords:** inositol phosphate, lithium, neurological disease, bipolar disorder, manic depressive illness, phosphatase, GD1, first gadolinium site, INPP1, inositol polyphosphate 1-phosphatase, IP, inositol phosphate, IP3, inositol 1,4,5-trisphosphate, MG1, magnesium metal site 1

## Abstract

Inositol polyphosphate 1-phosphatase (INPP1) is a prototype member of metal-dependent/lithium-inhibited phosphomonoesterase protein family defined by a conserved three-dimensional core structure. Enzymes within this family function in distinct pathways including inositide signaling, gluconeogenesis, and sulfur assimilation. Using structural and biochemical studies, we report the effect of substrate and lithium on a network of metal binding sites within the catalytic center of INPP1. We find that lithium preferentially occupies a key site involved in metal-activation only when substrate or product is added. Mutation of a conserved residue that selectively coordinates the putative lithium-binding site results in a dramatic 100-fold reduction in the inhibitory constant as compared with wild-type. Furthermore, we report the INPP1/inositol 1,4-bisphosphate complex which illuminates key features of the enzyme active site. Our results provide insights into a structural basis for uncompetitive lithium inhibition and substrate recognition and define a sequence motif for metal binding within this family of regulatory phosphatases.

Inositol phosphate (IP) signaling is critically important to cellular communication networks. Among the numerous lipid and soluble IP messengers, inositol 1,4,5-trisphosphate (IP_3_) is crucial for mediating the agonist-induced release of intracellular calcium stores (1, 2). Termination of IP_3_ signaling occurs, in part, through the action of IP kinases and phosphatases, the latter of which also function to replenish stores of inositol—metabolism reviewed in (3, 4). Links of inositol signaling to the pharmacology of lithium emerged when it was first reported that treatment of rats with lithium resulted in decreased brain inositol levels and led to the accumulation of inositol monophosphates and polyphosphates (5). Two IP phosphatase activities, inositol monophosphate phosphatase (IMPA1, IMPA2) and inositol polyphosphate 1-phosphatase (INPP1) were reported to be potently inhibited by lithium (6, 7, 8). Cloning and characterization of IMPA1 and INPP1 gene products confirmed they encoded metal-dependent lithium-inhibited enzymes with minimal sequence homology aside from a short six amino acid motif (9, 10).

Structural studies of IMPA1 and INPP1 illuminated that the “DPIDxT” six-amino acid motif anchor metal binding sites likely involved catalysis (11, 12). Remarkably, superimposition of alpha-carbons of these six residues of IMPA1 and INPP1 aligned 13 secondary structural elements of a 280 amino acid common core fold. Sequence comparison based on this three-dimensional alignment indicates they are prototype members of a small family of structurally conserved phosphatases, whose activities were not only limited to inositol signaling but also include regulation of gluconeogenesis and nucleotide metabolism (13). Upon completion of the human and mouse genome sequences, we now know that the family comprised of seven genes including IMPA1 and its ortholog IMPA2; INPP1; FBP1 and FBP2—fructose 1,6-bisphosphate phosphatases; BPNT1—adenosine 3’,5’ bisphosphate nucleotidase; and gPAPP—Golgi-resident 3’phosphoadenosine-5’phosphate phosphatase (14, 15). Of interest, all seven gene products are potently inhibited *in vitro* at or below therapeutic lithium concentrations (9, 10, 14, 15, 16, 17).

Detailed comparison of the structures of four members of the family suggests a common metal-dependent catalytic mechanism (11, 12, 18, 19). Conserved secondary structural elements comprise juxtaposed metal binding pockets and define a shared sequence pattern of D^54^…E^79^E^80^…D^153^PID^156^S^157^T^158^…D^317^ as shown for INPP1 (Fig. 1). Despite several reports that have defined the position of activating metal sites used for catalysis by this protein family (20, 21, 22, 23), understanding the mechanism of uncompetitive lithium inhibition has been challenging. Nonetheless, studies for three family members, IMPA1, yeast 3’-nucleotidase (Hal2p), and FBP1, have provided insights into catalytic metal orientation and effects of lithium on these configurations (12, 18, 20, 24, 25, 26). Other data include fluorescence quenching experiments in IMPA1 (27) and inference from coordination geometries of Li^+^ modeled into the active site of IMPA1 (21). In opposition to those data, support for Li^+^ inhibition at metal site 3 (MG3) include structural studies of the archael IMPA1/FBP1s implicating conformational changes associated in a loop region responsible for forming metal site 3 (28, 29). These results agree with earlier mutagenesis studies of human IMPA1 that showed reduced lithium sensitivity and the loss of inhibition by high concentrations of Mg^2+^ after mutation of a lysine residue found in the loop region that helps form metal site 3 (30).

**Figure 1:**
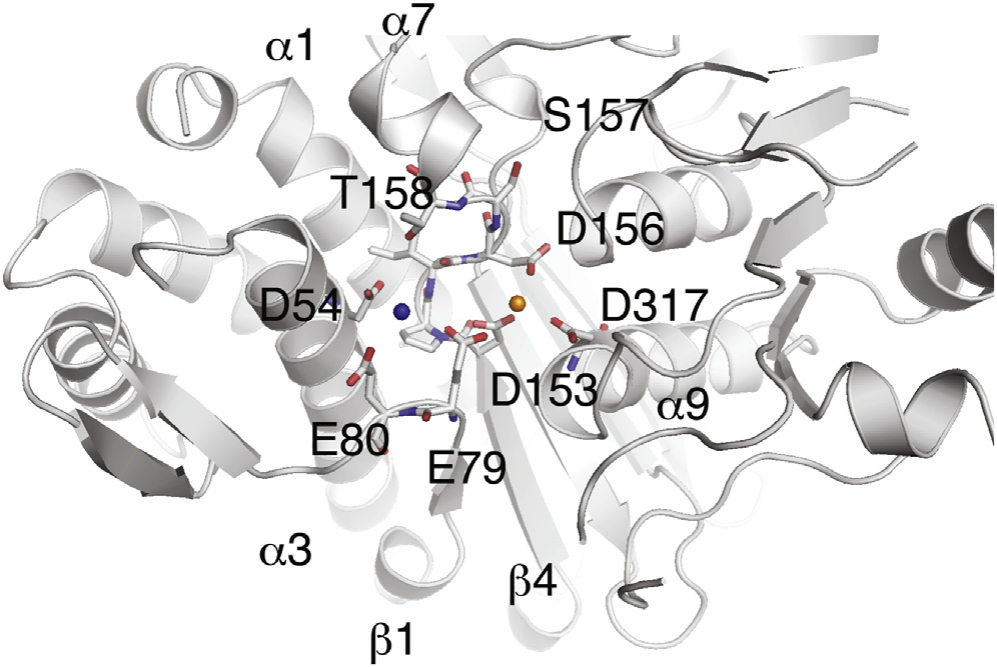
**INPP1: a prototype member of a family of metal-dependent lithium-inhibited phosphatases.** The metal-binding catalytic site and structure of INPP1 (PDB code: 1INP). Secondary structure elements are represented as a ribbon drawing, and the metal binding motif is shown as a stick figure. Two metal sites (MG3/GD3—blue and MG1/GD1—gold spheres) are anchored by labeled residues shared by members of the structurally conserved family of proteins. GD1, first gadolinium site; INPP1, inositol polyphosphate 1-phosphatase; MG1, magnesium metal site 1.

To further our understanding of lithium’s mechanism of action, here were report a systematic series of crystallographic studies of INPP1 that reveal the effect of lithium on the binding to the metal-enzyme complex when substrate or product is present. From these data, we infer where and how lithium binds in the INPP active site illuminating a structural basis for its uncompetitive pattern of inhibition. Additionally, we used this information to determine the structure of the INPP/substrate complex which provides additional mechanistic understanding of active site determinants and reaction mechanism.

## Results

### Gd^3+^ binds unequally to two metal sites in the absence of substrate or product

**Figure 2:**
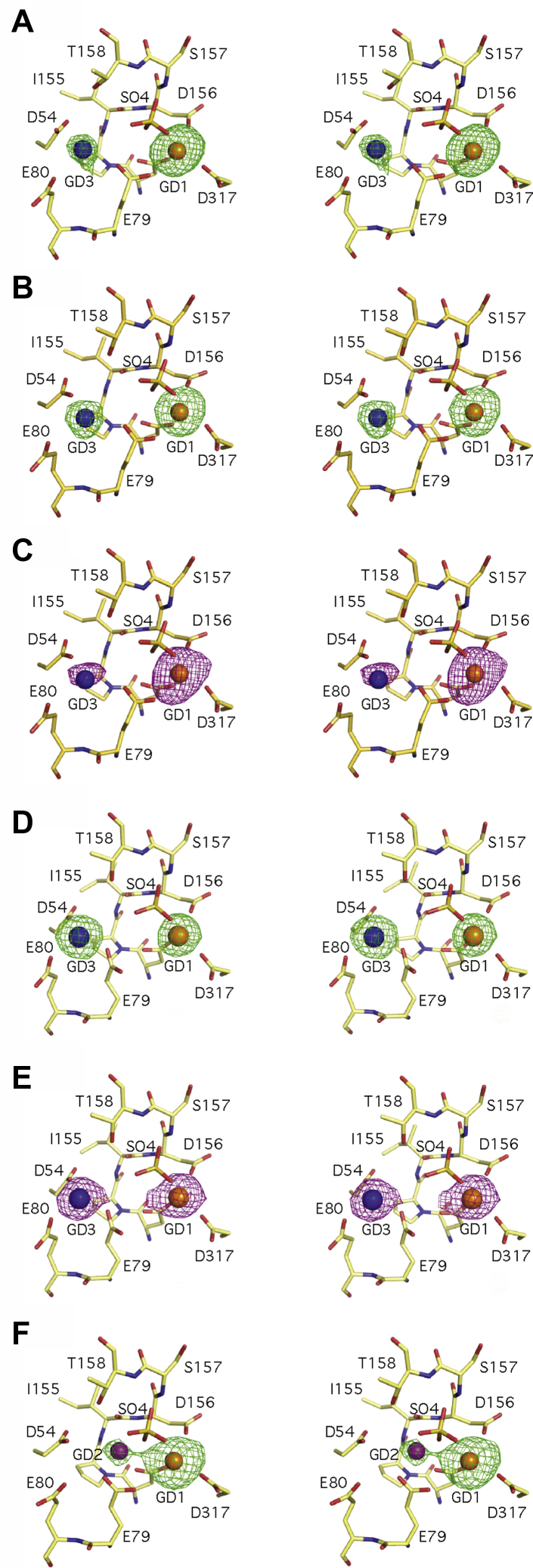
**Difference Fourier analysis of INPP1 complexes with gadolinium and/or lithium in the presence and absence of substrate.** Stereo-diagrams of INPP1 active site metals are shown for four separate experimental conditions. The protein component of the INPP1 active site is depicted as a stick diagram. Active site gadolinium metals are shown as spheres with the following numbering: GD1 (*orange*), GD2 (*purple*), and GD3 (*blue*). For each experimental condition, Fo-Fc difference density (*green mesh*, panels *A*, *B*, *D*, *F*) was calculated using phases from the protein component of native INPP1. Anomalous Fo-Fc difference density (*Magenta mesh*, panels C and E) was calculated in a similar manner. The ratio of individual Fo-Fc peak heights for each metal site (GD3:GD2:GD1) is listed. Metal sites with no detectable peak are listed as ND. Structures of INPP1 complexes were solved under the following conditions: (*A*) *Gd*^*3+*^*/Li*^*+*^; (*B*–*C*) *Gd*^*3+*^; (*D*–*E*) *Gd*^*3+*^*/Ins(1,3,4)P*_*3*_; and (*F*) *Gd*^*3+*^*/Ins(1,3,4)P*_*3*_*/Li*^*+*^. GD1, first gadolinium site; INPP1, inositol polyphosphate 1-phosphatase; MG1, magnesium metal site 1.

We initiated a series of crystallographic studies building upon the original structure determination of INPP1 by multiple isomorphous replacement (11). In these studies, the lanthanide metal gadolinium (Gd^3+^), a potent competitive inhibitor with respect to magnesium, was used in the presence of Li^+^ as one of the heavy atom derivatives and provides two key observations. The gadolinium ions found in the INPP1/Gd^3+^/Li^+^ structure bound predominantly to two sites in the INPP1 active site. Compared with the native INPP1/Mg^2+^/Li^+^ structure, the first gadolinium site (GD1) was well correlated with the position of the magnesium metal site 1 (MG1). The second gadolinium, however, was found 3.7 Å from the magnesium in site 2 (MG2) and was coordinated by a different set of protein and (presumably) solvent interactions. Given the anomalous differences of Gd^3+^, the identity of the metal in sites GD1 and the previously unseen metal site GD3 is unequivocal. Second, based upon difference Fourier analysis, the gadolinium metal density peaks were unequally occupied. Magnesium in the native structure had densities peaks of 4.4σ and 3.3σ in metal sites MG1 and MG2, respectively, giving a relative occupancy of 1.3. The gadolinium derivative showed a strong density peak (34.9σ) at GD1 and a relatively much weaker density peak (7.9σ) at GD3, giving a relative occupancy of the sites of 4.4 (Fig. 2*A*).

The crystals of INPP1 used for the initial structure determinations were grown in the presence of very high Li^+^ concentrations (11, 31). Thus, an explanation for the finding of metal mainly at site GD1 may be that Li^+^, for which the electron density is essentially invisible, may interfere with Gd^3+^ binding, either by directly competing for metal site GD3 or by inducing a conformational change thereby preventing binding. We first sought to rule out that the unequal occupancy of GD1 and GD3 was because of competition among the lesser electron dense metal. New crystals were grown under similar conditions with Gd^3+^ replacing Mg^2+^ but in the absence of Li^+^. Under these conditions, the electron densities for metal sites GD1 and GD3 gave a similar but less pronounced pattern of unequal occupancy (Fig. 2*B*). The Fo-Fc difference density gave peaks of 18.0σ and 10.5σ in metal sites GD1 and GD3, respectively, with a relative occupancy of 1.7. Similarly, the anomalous difference density showed peaks of 13.7σ and 5.7σ in metal sites GD1 and GD3, respectively, giving a relative occupancy of 2.4 (Fig. 2*C*). In the absence of substrate, both the Gd^3+^ and Gd^3+^/Li^+^ structures had gadolinium atoms that were poorly ordered in metal site GD3, refined to only half occupancy and had B-factors more than two-fold higher than the global average B-factor. This indicates in the absence of substrate or product, the addition of Li^+^ did not significantly affect the ratio of Gd^3+^ at sites GD1 and GD3, with metal binding strongly preferred at site GD1.

### Addition of substrate orders gadolinium at site GD3

The activity of INPP1 is cooperative with Mg^2+^ having a Hill co-efficient of 1.9 (7), supporting the idea that substrate binding alters Mg^2+^ affinity at additional metal sites. We tested the effects of substrate addition by co-crystallization of INPP1 with a 10X molar excess of Ins(1,3,4)P_3_ substrate and 2 mM inhibitory Gd^3+^. Although we could not confidently model the substrate in the resultant structure, metal sites GD1 and GD3 became equivalently occupied (Fig. 2*D*). Difference Fourier analysis showed densities peaks of 20.5σ and 20.0σ in metal sites GD1 and GD3, respectively, giving a relative occupancy of 1.0. Consistent with the Fo-Fc differences, anomalous differences peaks of 15.4σ and 14.0σ were calculated, giving a relative occupancy 1.1 (Fig. 2*E*). Whereas in the absence of substrate, the gadolinium in metal site GD3 were found at half occupancy and had B-factors greater than twice the average, and in the presence of substrate, the gadolinium in site metal site GD3 was well ordered, refined to full occupancy and had B-factors well below the global average.

### Lithium displaces metal in INPP1 at site GD3 in the presence of substrate

In the absence of substrate, the addition of lithium did not eliminate Gd^3+^ metal binding in INPP1. These data are consistent with an uncompetitive pattern of Li^+^ inhibition that predicts that substrate or product must be bound in order for Li^+^ inhibition to occur. We therefore tested the effect lithium addition in the presence of substrate. INPP1 was co-crystallized in the presence of 10X molar excess of Ins(1,3,4)P_3_ and 2 mM Gd^3+^, but also 200 mM Li^+^. As seen in Figure 2*F*, in the presence of substrate, no electron density peak was observed for Gd^3+^ at metal site GD3 with little change in density observed at metal site GD1. A 15.4σ difference density peak was found for metal site GD1, with no detectable peak for metal site GD3 indicating lithium displaces metal at site GD3 and that binding requires the presence of substrate. Interestingly, a 4.9σ difference density peak was found for gadolinium binding at GD2, the equivalent of MG2, indicating poorly ordered Gd^3+^ in that site (Fig. 2*F*). This provides further evidence Li^+^ does not displace metals at either sites GD1 or GD2.

### The active site metals of the lithium-inhibited phosphomonoesterase protein family

The native INPP1 structure was re-refined and agrees well with the original structure with an all-atom root mean square deviation of 0.25 Å. Importantly, the structural core and active site, including Mg^2+^ in metals sites MG1 and MG2, are essentially identical. In conjunction with the four INPP1-Gd^3+^ complexes presented here, at least three metal binding sites are seen in the active site of INPP1. Metal site MG1/GD1 has been observed in all structures, metal site MG2/GD2 has been observed in the Mg^2+^/Li^+^ and Gd^3+^/Li^+^/Ins(1,3,4)P_3_ structures, and the inhibitory metal site GD3 has been observed in the Gd^3+^, Gd^3+^/Li^+^, Gd^3+^/Ins(1,3,4)P_3_ structures.

**Figure 3:**
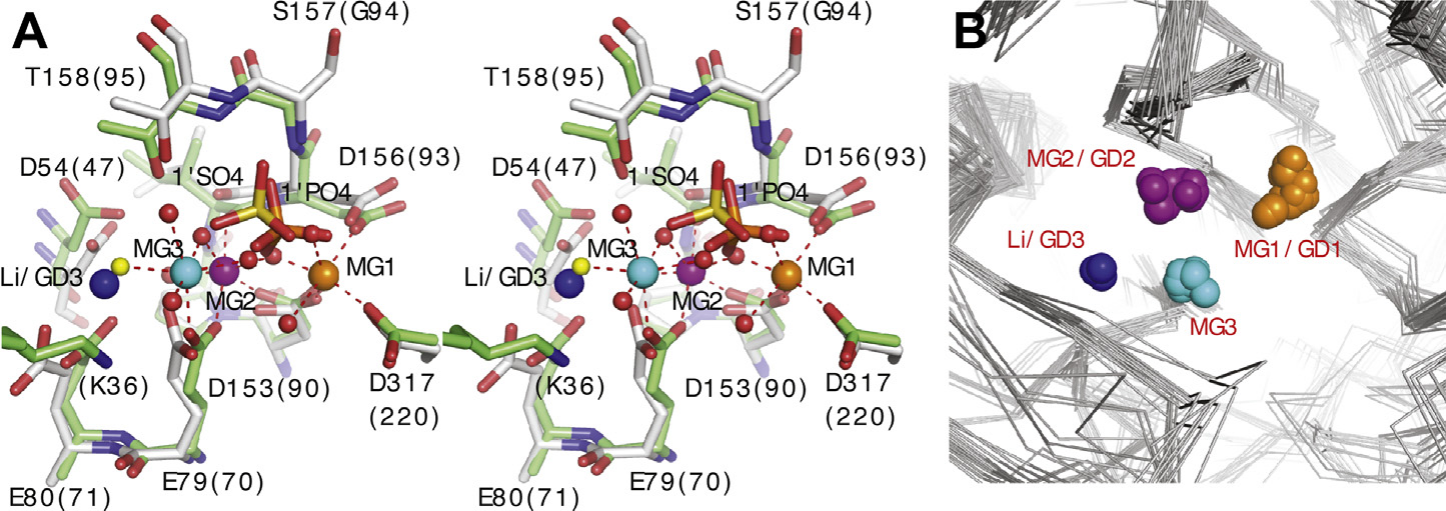
**Comparison of active sites from metal-dependent, lithium-inhibited phosphomonoesterases.***A*, stereo-diagram of the superimposed active sites of a high-resolution IMPA1-Mg^2+^ complex (PDB ID 2BJI) and INPP1-Gd^3+^/Ins(1,3,4)P_3_ complex. The protein component of the INPP1 (*gray*) and IMPA1 (*green*, numbering in parenthesis) complexes are shown as *sticks*. Metals atoms are shown as large spheres for the MG1/GD1 (*orange*), MG2/GD2 (*purple*), MG3 (*cyan*), and GD3 (*blue*) sites. Water molecules, shown as *small red spheres*, are taken from the IMPA1 structure. *Red dashed lines* depict the coordination of the activating Mg^2+^ metals of IMPA1. The *small yellow sphere* represents a water atom that coordinates metal site MG3 in the IMPA1 structure. This water also corresponds to lithium binding site (GD3) in the INPP1 complexes. A sulfate ion from the INPP1 complex (1’ SO^−^_4_, *yellow sticks*) is well correlated with the 1-position PO^−^_4_ of Ins(1)P_1_ from a IMPA1-Ins(1)P_1_ complex (PDB=1IMA; *orange sticks*). The inositol ring of Ins(1)P_1_ is omitted for clarity. *B*, the superposition of structures from metal-dependent, lithium-inhibited phosphomonoesterase family members reveals that active site metals cluster around defined sites. Structures of family members that contain at least one active site metal were obtained from the Protein Data Bank (PDB) and aligned using the active site residues. Representative family members and their structures include FBP: fructose bisphosphatase (PDBs = 1G0H, 1G0I, 1FPI, 1FPJ, 1FPK, 1FPL, 1CNQ, 1EYI, 1EYJ, 1EYK), FBP-IMP: archael hybrid fructose and inositol phosphatase (PDBs = 1LBX,1DK4), IMP: inositol monophosphatase (PDBs = P2BJI, 1IMA, 1IMB, 1IMC, 1IMD, 1IME, 2HHM, 2CZI, 1AWB, 2P3N), BPNT: bisphosphate nucleotidase (PDBs = 1GQX, 1KAI, 1K9Y, 1K9Z, 1KA0, 1JP4), and INPP1: inositol polyphosphate phosphatase (1Inp, current study). The protein Cα backbone is depicted as a gray ribbon. The active site metals are shown as spheres and are numbered according to INPP1 convention: MG1/GD1 (*orange*), MG2/GD2 (*purple*), MG3 (*cyan*), and Li/GD3 (*blue*). GD1, first gadolinium site; IMPA, inositol monophosphate phosphatase; INPP1, inositol polyphosphate 1-phosphatase; MG1, magnesium metal site 1.

The INPP1 GD3 metal site is mediated by two protein–metal contacts to residues D54 and E80. In other family members, there appears to be only one protein-mediated contact to the activating metal site MG3. The other five coordinating ligands of MG3 are water molecules giving this site a unique plasticity that could accommodate different coordination geometries (Fig. 3*A*) (21). The lithium sensitive GD3 binding site corresponds to one of the five waters that coordinate the MG3 metal site (Fig. 3*A*). The GD3/coordinating water site is also in close proximity to a lysine residue in IMPA1 that when mutated dramatically reduces lithium sensitivity and renders the enzyme resistant to Mg^2+^ inhibition at high concentration (30), further suggesting a role for this site in lithium inhibition.

An overlay of the INPP-metal complexes with structurally related lithium-inhibited phosphomonoesterase proteins with one or more active site metals reveals that their active site metals cluster around defined sites (Fig. 3*B*) (11, 12, 18, 21, 22, 23, 24, 25, 26, 28, 29, 32, 33, 34, 35, 36). The positions of metal sites corresponding to INPP1 MG1/GD1 and MG2/GD2 are well conserved with an average pairwise distance for 26 and 27 metal atoms of 0.8 Å and 0.7 Å, respectively, and represent two of three activating metal sites. In contrast, the lithium-sensitive and inhibitory gadolinium metal binding site has only yet been observed in INPP1. Members of this family conserve a third activating metal site (MG3) (Fig. 3*B*). Notably, this site is distinct from the Li/GD3 binding site observed in the INPP1 structures, and its position away from putative water nucleophile may explain the inhibitory effect these metals exert.

### Lithium inhibition studies of INPP1 wild-type *versus* D54A mutant

**Figure 4:**
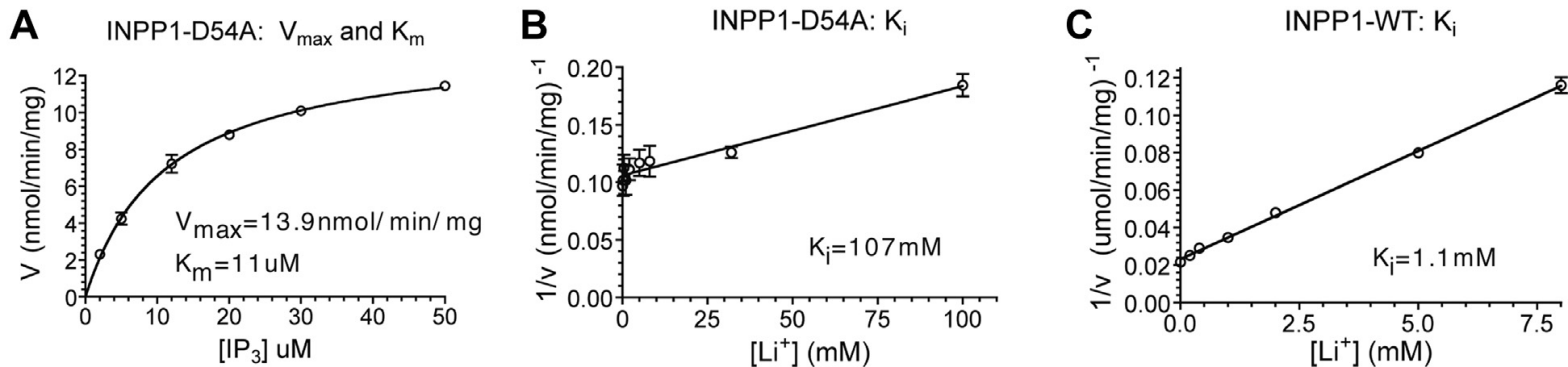
**Lithium inhibition of the INPP1 and INPP1**^**D54A**^**lithium binding site mutant.***A*, specific activity for the INPP1^D54A^ catalyzed hydrolysis of Ins(1,3,4)P_3_. Enzyme velocity (nmol/min/mg) is plotted as a function of substrate concentration ([I(1,3,4)P_3_]). The V_max_ and K_m_ of the reaction are indicated. *B*–*C*, dixon plots of 1/v *versus* [Li^+^] for the hydrolysis of Ins(1,3,4)P_3_ by the INPP1D^54^A mutant and INPP1 wild-type enzyme. Respective K*i* values are indicated. INPP1, inositol polyphosphate 1-phosphatase.

Based on our crystallographic studies, we found that the lithium-sensitive, inhibitory metal binding site was coordinated by the carboxylates of two conserved residues, D54 and E80. Residue D54 was mutated to alanine, and we kinetically examined the effect of Li^+^ inhibition upon the wild-type and mutant enzymes. Mutant INPP1^D54A^ exhibited a 4300-fold inactivation relative to the wild-type enzyme with a V_max_ of 13.9 nmol/min/mg compared with 59.7 μmol/min/mg for the wild-type enzyme (Fig. 4*A*) (31). While a large change was observed for V_max_ values, the substrate affinity appeared to be relatively unaltered with K_m_ values of 11 μM to 31 μM for the D54A mutant and wild-type enzymes (Fig. 4*A*) (31). Although the D54A mutation does not significantly alter Km, there was a large effect on K_i_. Figure 4, *B*–*C* show linear plots of 1/v *versus* [Li^+^], for the hydrolysis of Ins(1,3,4)P_3_ by the D54A mutant and wild-type enzyme. As seen in Figure 4*B*, the x-intercept of this plot (−K_i_ (1 + K_M_/[Ins(1,3,4)P_3_])) gives a K_i_ value of 107 mM for the D54A mutant. This represents a >100-fold decrease in Li^+^ sensitivity for the D54A mutant compared with the wild-type INPP1 (Fig. 4*C*). The results from the kinetic studies corroborate our structural studies that the site of Li^+^ inhibition in INPP1 is located at metal site GD3.

### Active site sulfate/phosphates found in INPP1

Two sulfate and/or phosphate ions were found in the active sites of the INPP1 structures. In the Gd^3+^ and Gd^3+^/Li^+^ structures, the sulfates originated presumably as the Gd^3+^ counter-ion from the Gd_2_(SO_4_)_3_ used in the crystallization conditions. In the Gd^3+^/Ins(1,3,4)P_3_ and Gd^3+^/Li^+^/Ins(1,3,4)P_3_ structures, the ion could either be a phosphate from the Ins(1,3,4)P_3_ substrate molecule or a sulfate from the Gd_2_(SO_4_)_3_ in the crystallization solutions. We were unable to distinguish these possibilities. However, as seen in Figure 3*A*, a sulfate (or phosphate) in the INPP1 structures correlates well with the 1-position phosphate of Ins(1)P_1_ observed in the IMPA1/Ins(1)P_1_ complex (25), and the inositol ring from this structure is omitted for clarity. The second sulfate is located near the 4-position of the inositol ring from Ins(1)P_1_ and was therefore labeled 4’SO^−^_4_. While we did not observe the Ins(1,3,4)P3 substrate, we hypothesize the sulfates and/or phosphates seen in the INPP1 structures represent the 1- and 4-PO_4_ positions that would be found with ordered substrate or reaction product.

### Structure determination INPP1^D54A^ metal and substrate complexes

We next attempted to directly examine substrate binding by INPP1. As mutation of D54 results in loss of INPP1 activity without significantly changing the substrate affinity, we hypothesize that the D54A mutant can be utilized to trap the substrate in INPP1. To rule out the possibility of unanticipated conformational changes, we first determined the structure of INPP1^D54A^ in the presence of calcium. One calcium ion at metal site 1 is found in the INPP1^D54A^/Ca^2+^ structure, alongside two sulfate ions similar to those found in Gd^3+^ structures. The INPP1^D54A^/Ca^2+^ structure reveals that D54A mutation does not cause obvious conformational changes, exhibiting an root mean square deviation of 0.6 Å between main-chains of the mutant and the wild-type protein

**Figure 5:**
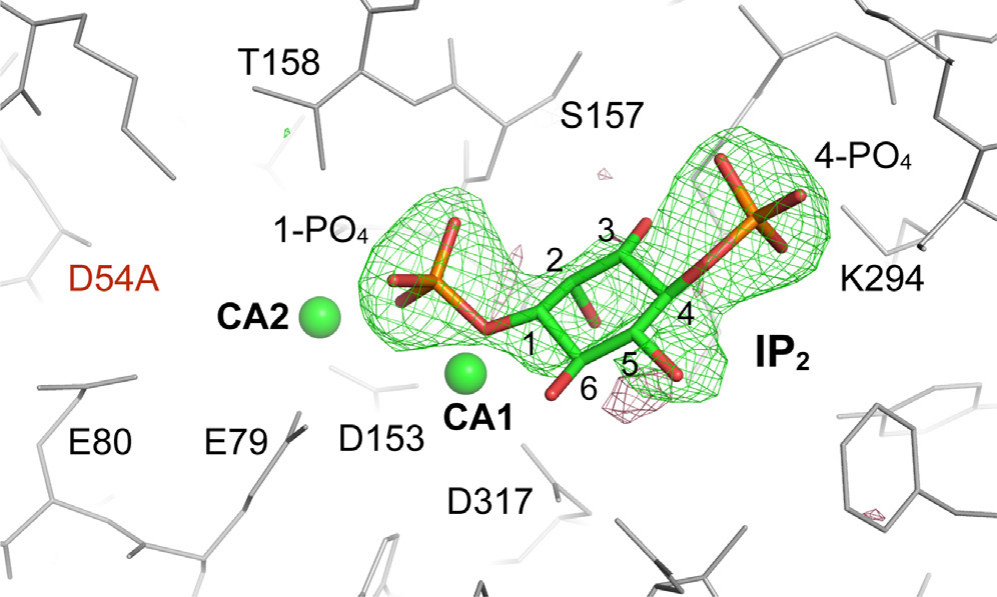
**Ins(1,4)P**_**2**_**recognition by INPP1**^**D54A**^**.** Diagram of the active site in the INPP1^D54A^/Ca^2+^/Ins(1,4)P_2_ structure. Ins(1,4)P_2_ is shown in sticks. Two calcium ions (CA1 and CA2) are shown as spheres. Electron density attributed to substrate binding (*green mesh*) represents the mFo-DFc omit map calculated with phases derived from the refined structure with Ins(1,4)P_2_ omitted. Both positive (*green mesh*) and negative (*red mesh*) densities are displayed at a contour level of 3.0σ. INPP1, inositol polyphosphate 1-phosphatase.

We next soaked the INPP1^D54A^/Ca^2+^ crystals with the Ins(1,4)P_2_ substrate. Indeed, the D54A mutant led to structure determination of the substrate bound INPP1^D54A^/Ca^2^/Ins(1,4)P_2_ complex. Two calcium ions (CA1 and CA2) are found at metal sites 1 and 2. We did not observe any metal binding at MG3/GD3 in the D54A mutant. The mFo-DFc omit map of the Ins(1,4)P_2_ substrate exhibits continuous electron density that agrees with the shape of Ins(1,4)P_2_ in the active site of the enzyme (Fig. 5). Substrate binding is largely contributed by extensive interactions involving the 1-PO_4_ and 4-PO_4_ groups. For 1-PO_4_, one oxygen serves as ligand for both CA1 and CA2; another oxygen forms hydrogen bonds with main chain amide groups of S157 and T158 residues residing in the “DPIDST” motif. These 1-PO_4_ mediated interactions are reminiscent of those observed in the IMPA1/Ins(1)P_1_ structure (25). The 4-PO_4_ group forms hydrogen bonds with side-chain of K294 and multiple main chain amide groups involving E269, G290, and A291. Furthermore, binding of Ins(1,4)P_2_ is reinforced by interactions between the inositol ring and surrounding residues. Together, the INPP1^D54A^/Ca^2^/Ins(1,4)P_2_ complex structure reveals the molecular basis for substrate recognition by INPP1.

## Discussion

**Figure 6:**
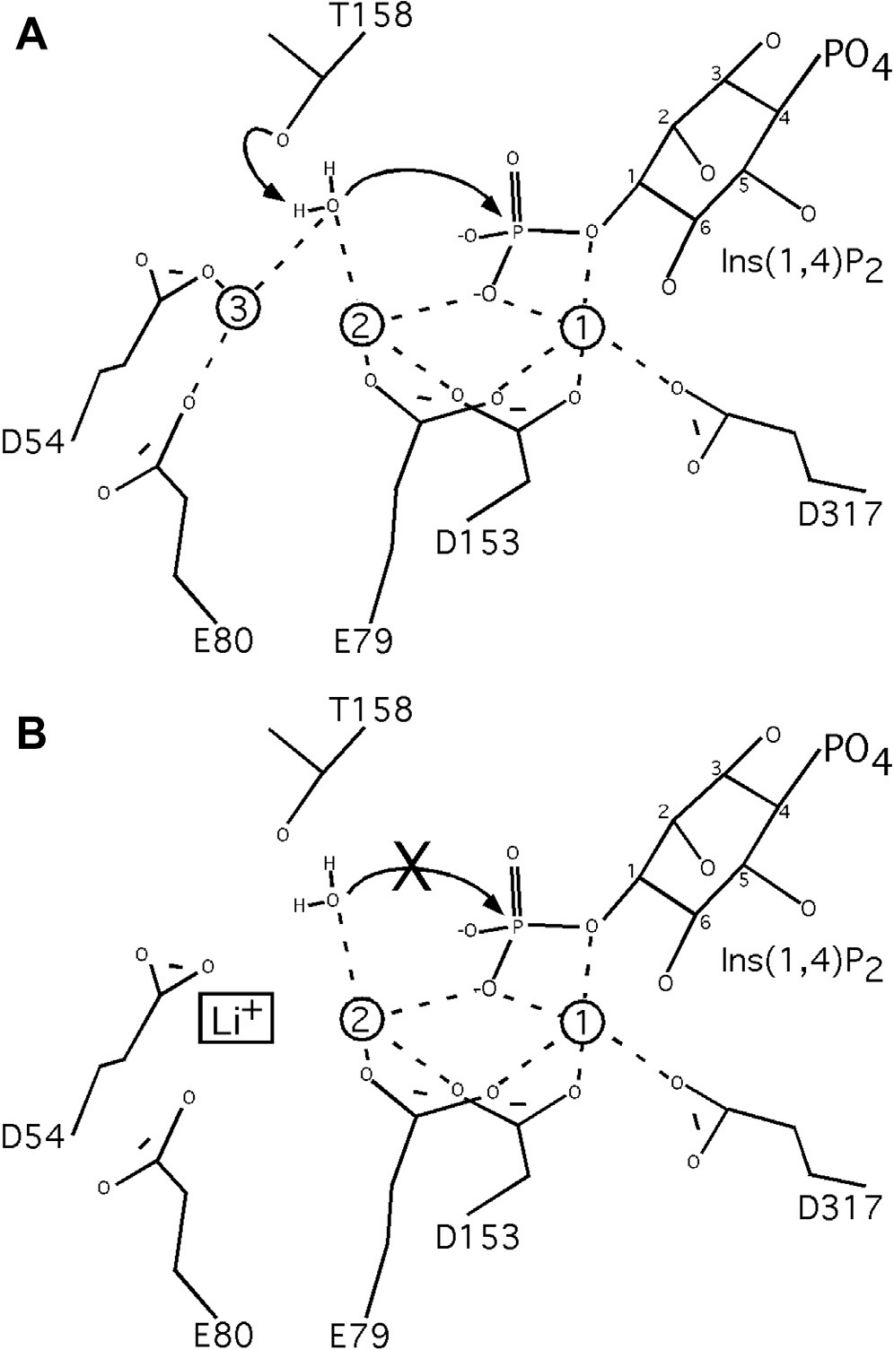
**Mechanistic model for the metal-dependent hydrolysis of phosphomonoester bonds by INPP1.***A*, three metal sites in the active site of INPP1 are shown as hollow spheres labeled 1, 2, and 3—derived from various Mg^2+^, Ca^2+^, and Gd^3+^ structural data. Mg^2+^ in metal sites 2 and 3 coordinate a nucleophilic water that we postulate is activated by threonine residue 158 for an inline attack on 1’PO_4_ of Ins(1,4)P_2_. Metal site 2 is coordinated by three protein-mediated contacts to residues E79, D153, and carbonyl oxygen of I155. Metal site 3 is coordinated by two protein-mediated contacts, D54 and E80. Three conserved aspartic acids, D153, D156 (omitted for clarity), and D317, compose metal site 1, from which Mg^2+^ coordinates the ester oxygen and helps to stabilize the developing negative charge, as the phosphate ester is cleaved. *B*, uncompetitive lithium inhibition of the INPP1 reaction may occur through its binding at metal site 3, possibly interfering with nucleophilic attack by water or through altering substrate reloading/binding. INPP1, inositol polyphosphate 1-phosphatase.

In this report, we provide evidence for how lithium binds to and inhibits the inositide phosphatase INPP1, a prototype member of a family of structurally conserved family of enzymes whose activities are potently inhibited by lithium. Using X-ray crystallographic analysis of several INPP1 complexes in the presence and absence of substrate and/or lithium, we define structural bases for (1) the role of a conserved sequence motif responsible for lithium’s effect; (2) lithium’s uncompetitive pattern of inhibition; and (3) Ins(1,4)P_2_ substrate binding. Our systematic approach provides snapshots of how substrate and/or product influence metal binding as well as outlining critical residues that mediate the effects. Overall, we summarize our model using the schematic (Fig. 6*A*). INPP1 catalyzes the metal-dependent hydrolysis of the phosphomonoester bond from the 1 position phosphate of Ins(1,4)P_2_ or Ins(1,3,4)P_3_ to yield inositol 4-phosphate and inositol 3,4-bisphosphate, respectively (6, 7, 37, 38). Consistent with other family members for which a detailed kinetic mechanism has been described (21, 22, 23), we postulate a nucleophilic water coordinated by Mg^2+^ in metal sites 2 and 3 is activated for an inline attack on the labile phosphate by a conserved threonine residue, T158. Metal site 2 is coordinated by three protein-mediated contacts to residues E79, D153, and I155. Metal site 3 is coordinated by two protein-mediated contacts, D54 and E80. Three conserved aspartic acids, D153, D156, and D317, compose metal site 1, from which Mg^2+^ coordinates the ester oxygen and helps to stabilize the developing negative charge, as the phosphate ester is cleaved.

We specifically chose to use Gd^3+^, an electron dense lanthanide, as a proxy for catalytically required Mg^2+^. Despite gadolinium’s coordination ligand number of 6 to 9, as compared with 4 to 6 for magnesium, we observed that the positioning of three Gd^3+^ sites identified are similar to those observed or proposed for Mg^2+^. In the absence of substrate, co-crystallization of INPP1 with Gd^3+^, a competitive inhibitor of Mg^2+^, resulted in preferred metal binding to site GD1 to GD3. Addition of Li^+^ in the absence of substrate did not significantly affect the ratio of Gd^3+^ at these sites. Co-crystallization with Gd^3+^ but in the presence of substrate (I(1,3,4)P_3_) resulted in equivalent occupancies of metal sites GD1 and GD3 consistent with the hypothesis that substrate binding alters affinity at metal site GD3 by contributing an additional coordinating ligand through phosphate oxygen. The observation occupancy at a second/third site is also consistent with previous kinetic studies which demonstrated metal binding in INPP1 is cooperative with a Hill co-efficient of 1.9 which predicts if metal site 1 is predominantly bound in the absence of substrate, that an additional two metals are bound cooperatively upon substrate binding (7). It is noteworthy that the addition of substrate resulted in a significant change in the ratio and intensity of electron density peaks such that metal sites equivalent occupancies of sites GD1 and GD3, each having a tremendous signal exceeding 20 sigma. Anomalous signals confirm this is because of metal binding.

An important mechanistic insight gleaned from our studies relates to the constitutive occupancy of metal at site 1. Regardless of experimental condition, the INPP1 metal site MG1/GD1 is found fully occupied and is accompanied by a strong, relatively unchanged, electron density peak. This is in stark contrast to what is observed at metal site GD3, which undergoes dramatic changes in the electron density signal depending on the addition of substrate and lithium. Furthermore, mechanistically, MG1/GD1 metal binding does not appear cooperative with respect to substrate binding nor is susceptible to displacement by lithium under any of the conditions examined. Our conclusions of a three-metal–assisted catalytic mechanism for INPP1 are consistent with proposed models for several other members of the Mg^2+^-dependent/Li^+^-sensitive phosphoric monoester hydrolase family, including IMPA1 (20, 21), FBP1 (23), and FBP1/IMPA1 (22). Our work supports an evolutionary conservation of the enzymology of this important family.

The uncompetitive pattern inhibition of INPP1 by Li^+^ predicts that substrate or product must be bound in order for Li^+^ inhibition to occur. As discussed earlier, addition of Li^+^ alone had minimal observable effect on the electron density observed for Gd^3+^ at any of the three sites. In dramatic contrast, co-crystallization of INPP1 with Gd^3+^, I(1,3,4)P_3_ and Li^+^ resulted in a stunning complete loss of electron density at metal site GD3 (no observable density even at below 1 sigma) with little change in density at site GD1 (remained above 20 sigma). Because we did not observe significant changes in the side-chain geometry for residues that coordinate the metal binding sites, we infer the parsimonious explanation is lithium replaces gadolinium at metal site 3. A partially ordered Gd^3+^ was found in metal site GD2 in the substrate-bound, lithium-treated structure and importantly excludes this metal site as the lithium-binding site. This hypothesis is supported by our kinetic analysis of the INPP1^D54A^ mutant protein which demonstrated a 100-fold reduction in the potency of lithium inhibition (*K*_i_ went from 1 mM to over 100 mM with no significant change in *K*_m_).

Given the systematic structural approach utilized, our work helps to unambiguously define a basis for lithium’s uncompetitive pattern of inhibition through its effects at metal site 3 (Fig. 6*B*). It is important to note, studies of IMPA1 indicate potential differences for the precise site at which lithium acts among family members (21). It is possible that given the high concentrations of metals used in different studies may account for subtle differences, for example, the inhibitory metals gadolinium and lithium bound in an INPP1 site that corresponds to a water molecule used for the coordination of MG3 (Fig. 3*B*) (21). Therefore, while some support exists for lithium inhibition at MG1 (12, 18, 21, 24, 27), our data are most consistent with lithium binding disrupting the coordination of metal site MG3 (GD3). The determination that lithium binds to a site responsible for the coordination of MG3 is corroborated by several previous studies. Crystal structures of *Archael* IMPA1/FBP1s show that the conformation and length of a loop region containing residues that help form metal sites GD3 and MG3 directly correlates to the sensitivity to Li^+^ inhibition (28, 29). Further, mutagenesis of a conserved IMPA1 lysine residue in close proximity to this site results in reduced sensitivity to lithium inhibition and resistance to inhibition at high Mg^2+^ concentrations (30). Because these observations support our findings and given the remarkable conservation of their active sites, the results presented here may extend to other family members.

## Experimental procedures

### Protein expression, purification, and crystallization

Recombinant INPP1 was prepared as previously described (31). Briefly, cDNA encoding bovine INPP1 was transfected into Sf9 insect cells *via* Baculovirus infection and grown until enzyme levels were >25 mg/l of medium. Over-expressed protein was concentrated, dialyzed, and sequentially passed over *diethylaminoethyl cellulose* and phenyl HPLC columns and was purified to homogeneity as judged by SDS-PAGE analysis. The INPP1^D54A^ mutant used for the kinetic and structural studies was over-expressed and purified to homogeneity as described for wild type.

INPP1 tetragonal-shaped crystals amenable to diffraction studies were grown by the hanging drop vapor diffusion method on silanized glass covers slips at 20 °C and grew with dimensions routinely exceeding 0.1 × 0.1 × 0.5 mm. Gd^3+^, a competitive inhibitor of Mg^2+^, has a useful anomalous absorption edge at the CuKα wavelength and therefore was used in the co-crystallization or soaking conditions. Four separate conditions were used to produce crystals in the course of these studies. In each case, purified recombinant bovine INPP1 from pooled Phenyl HPLC fractions was concentrated in a Centricon-30 concentrator, then exchanged with buffer 50 mM Tris HCl (pH 6.5) five times (approximately 10,000-fold buffer exchange). For crystallization, 3 μl of 13 mg/ml INPP1 was added to 3 μl of reservoir solution: (1) -*Mg*^*2+*^/*-Li*^*+*^*/-Ins(1,3,4)P*_*3*_*/+Gd*^*3+*^ (6WRO): Reservoir solution consisted of 10% PEG 8000, 80 mM Bis-Tris, pH 5.25, 2 mM Gd_2_(SO_4_)_3_; (2) *+Mg*^*2+*^*/+Li*^*+*^*/-Ins(1,3,4)P*_*3*_*/soak Gd*^*3+*^ (6WRR): Reservoir solution consisted of 13% PEG8000, 200 mM Li_2_SO_4_, and 100 mM Tris, pH 6.3. Upon reaching maximum size after ∼5 days, a Gd^3+^ heavy atom derivative was prepared by adding 0.5 μl of 17.5 mM Gd_2_(SO_4_)_3_ in reservoir solution to 3 μl hanging drops (2.5 mM final Gd^3+^) containing native crystals for 48 h; (3) -*Mg*^*2+*^/-*Li*^*+*^*/+Ins(1,3,4)P*_*3*_*/+Gd*^*3+*^ (6WRY): Reservoir solution consisted of 10% PEG 8000, 80 mM Bis-Tris, pH 5.25, 2 mM Gd_2_(SO_4_)_3_, and 1 mM Ins(1,3,4)P_3_; and (4) -*Mg*^*2+*^/*+Li*^*+*^*/+Ins(1,3,4)P*_*3*_*/+Gd*^*3+*^ (6X25): Reservoir solution consisted of 10% PEG 8000, 80 mM Bis-Tris, pH 5.25, 2 mM Gd_2_(SO_4_)_3_, 1 mM Ins(1,3,4)P_3_, and 200 mM Li_2_SO_4_.

Purified recombinant bovine INPP1^D54A^ was concentrated in a Centricon-30 concentrator, then exchanged with buffer 50 mM Tris HCl (pH 6.5) five times (approximately 10,000–fold buffer exchange). Enzyme concentration was determined using the Bio-Rad protein dye assay. INPP1^D54A^ yielded crystals using PEG6K as a precipitant. Briefly, 5 to 10 mg/ml INPP1^D54A^ in 50 mM Tris HCl (pH 6.5) was mixed with an equal volume of precipitant A—0.1 M Tris HCl pH 5.5, 40 to 43% saturated (NH_4_)_2_SO_4_, 2 mM Gd_2_(SO_4_)_3_—or precipitant B—0.1 M sodium citrate pH 4.3, 16 to 18% PEG6K, 5 mM CaCl_2_—and subjected to hanging drop vapor diffusion trials at 17 °C. Crystals typically appeared after 3 to 4 days and stopped growing after 1 week. Enzyme substrate complexes were formed *in situ* by soaking mutant crystals which had been washed 3 times in precipitant A or B solution in 50 to 200 μM of substrate Ins(1,4)P_2_ for 1 to 3 h.

### Data collection

INPP1 diffraction data from capillary mounted crystals were collected on dual Xuong-Hamlin Mark II multiwire area detectors (39) equipped with helium boxes using CuKα X-rays generated by a Rigaku RU200 rotating anode. As was the case with the original INPP1 data sets (11), several data sets had to be reindexed from *h,k,l* to *h, -k, -l* to keep indexing consistent. This problem arises from the physical orientation along the C axis of crystals with P4 point groups. Inverse beam experiments were used to collect anomalous data by complementing ϕ,χ orientations with ϕ + 180°, -χ orientations. Data were reduced, merged, and scaled using the area detector software (40). Data from the inverse beam experiments were reduced without merging the Bijvoet pairs and were subsequently used to calculate the anomalous signal component. In preparation for structure solution and refinement, reflection files were converted to mtz format and assigned a conserved R_free_ set using the program f2mtz (41).

INPP1^D54A^ and substrate complex diffraction data were collected on an R-Axis IIc image plate detector using monochromatic Cu K_α_ X-rays (λ = 1.5418 Å) at 4 °C to prevent crystal decay and were processed using DENZO and SCALEPACK (42).

### Re-refinement of the native INPP1 structure

The native INPP1 structure (1INP) was solved and refined before the use of R_free_ test data sets (43). We therefore sought to re-examine the INPP1 model in context of the current studies by taking advantage of a R_free_ test set and modern structure refinement software. The original reflection file for the native INPP1 (11) was converted to mtz format and assigned a conserved R_free_ for re-refinement. The R_free_ set was taken an INPP1-IP_2_ complex that crystallized in the same space group and unit cell dimensions. The native INPP1 data with conserved R_free_ set were solved by molecular replacement with Phaser (44) using the protein component of the INPP1–IP_2_ complex. Model building was completed with iterative rounds of building in Coot (45) using difference Fourier maps and automated refinement with an MLF target in Refmac5 (46), including isotropic B-factor and TLS refinement (47).

### Structure determination and refinement of INPP1 and INPP1^D54A^ complexes

The structures of INPP1 co-crystallized or soaked in the presence of Gd^3+^, Gd^3+^/Li^+^, Gd^3+^/Ins(1,3,4)P_3_, and Gd^3+^/Li^+^/Ins(1,3,4)P_3_ were solved by direct Fourier transform in Refmac (46) using phases derived from the protein component of the re-refined native INPP1 structure. Each structure refined independently against data collected to 3.00 Å, 2.50 Å, 2.80 Å, and 3.20 Å, respectively. Model building was completed with iterative rounds of building in Coot (45) using difference Fourier maps and automated refinement with an MLF target with Refmac5 (46), including isotropic B-factor refinement for the Gd^3+^/Li^+^ data and overall B-factor refinement for the Gd^3+^, Gd^3+^/Ins(1,3,4)P_3_, and Gd^3+^/Li^+^/Ins(1,3,4)P_3_ data. Anomalous difference maps for the Gd^3+^ and Gd^3+^/Ins(1,3,4)P_3_ data were calculated in fast fourier transfaorm (41) using phases derived from re-refined native INPP1 structure and were used to verify the placement and identity of the Gd^3+^ atoms. Structure validation for all structures was performed using MolProbity (48). Molecular graphics and structural alignments were created with Pymol (http://www.pymol.org). Data collection and refinement statistics are given in Table 1.

**Table 1:** Data collection and refinement statistics for INPP1 structures

Diffraction data	+ Mg^2+^ + Li^+^	+ Gd^3+^ - Li^+^	+ Mg^2+^ + Li^+^ soak Gd^3+^	+ Gd^3+^ - Li^+^ Ins(1,3,4)P_3_	+ Gd^3+^ + Li^+^ Ins(1,3,4)P_3_
PDB code	1INP	6WRO	6WRR	6WRY	6X25
Space group	P4_1_	P4_1_	P4_1_	P4_1_	P4_1_
*a = b, c* (Å)	51.6, 143.3	51.8, 143.2	51.5, 142.9	52.1, 143.1	51.9, 142.8
Wavelength (Å)	1.5418	1.5418	1.5418	1.5418	1.5418
Resolution limit (Å)^a^	2.30	3.00	2.50	2.80	3.20
Last shell	2.30–2.48	3.00–3.23	2.50–2.69	2.80–3.02	3.20–3.45
Unique Reflections	17,652	12,719^b^	26,197^b^	15,787^b^	11,374^b^
Completeness (%) (last shell)	99.3 (99.6)	78.6 (70.0)	96.0 (95.6)	79.0 (70.0)	85.0 (74.9)
Average I/σ_I_	10.5	3.5	10.8	4.2	3.8
Redundancy	3.6	1.3	3.4	1.5	2.1
R_sym_^c^ (%) (last shell)	5.0 (14.0)	8.0 (15.9)	5.1 (17.2)	6.1 (12.7)	10.3 (19.3)
**Crystallographic refinement**					
Resolution range (Å)	22.80–2.30	8.30–3.00	15.45–2.50	6.95–2.80	10.22–3.20
Reflections	16,006	6935	12,372	7750	5527
Rms deviation from ideality					
Bond lengths (Å)	0.015	0.006	0.008	0.005	0.007
Bond angles (°)	1.501	0.817	1.056	0.757	1.040
Rotamer outliers	3.57%	1.09%	2.90%	1.08%	0.36%
Ramachandran					
Outliers	0.0%	0.0%	0.0%	0.0%	0.0%
Allowed	100%	100%	100%	100%	100%
Favored	96.9%	98.1%	98.1%	98.4%	96.5%
R value^d^ (%)	16.4	19.3	16.3	20.8	19.9
R_free_ (%)	21.4	23.6	20.5	24.9	24.6

INPP1, inositol polyphosphate 1-phosphatase; PDB, Protein Data Bank.

a Resolution limit was defined as the highest resolution shell, where the average I/σ_I_ was >1.2 and R_sym_ <20%.

b Bijvoet pairs unmerged.

c R_sym_ = ∑_hkl_∑_i_∣I_i(hkl)_ − <I_(hkl)_>∣/∑_hkl_∑_I_I_(hkl)_.

d R = ∑∣F_o_ − F_c_∣/∑F_o_. 5% of reflections were used to calculate R_free_.

The structures of INPP1^D54A^/Ca^2+^ and INPP1^D54A^/Ca^2+^/Ins(1,4)P_2_ were solved by direct Fourier transform in Refmac (46) using phases derived from the protein component of the re-refined native INPP1 structure. Difference Fourier map with (|Fo|-|Fc|)e^iϕcalc^ coefficients was then calculated to check the electron density around the side-chain of carboxyl group of D54. The negative density around the carboxyl group of D54 confirmed the nature of the mutation. For the INPP1^D54A^/Ca^2+^/Ins(1,4)P_2_ structure, programs from the CCP4 suite (41) were used to calculate sigma-weighted difference Fourier maps with phases derived from the INPP1^D54A^/Ca^2+^ structure. Substrate Ins(1,4)P_2_ was initially modeled to the positive density using Turbo/Frodo program. Model building was completed with iterative rounds of building in Coot (45). Refinement of INPP1^D54A^/Ca^2+^ and INPP1^D54A^/Ca^2+^/Ins(1,4)P_2_ was carried out in both reciprocal space and real space using Phenix (49) with secondary structure restraints. Data collection and refinement statistics are given in Table 2.

**Table 2:** Data collection and refinement statistics for INPP1^D54A^ structures

Diffraction data	INPP1^D54A^: Ca^2+^	INPP1^D54A^: Ca^2+^+Ins(1,4)P_2_
PDB code	7KIO	7KIR
Space group	P4_1_	P4_1_
*a = b, c* (Å)	51.45, 143.12	51.44, 142.94
Wavelength (Å)	1.5418	1.5418
Resolution (Å)^a^	28.9–2.40	35.3–2.60
Last Shell (Å)	2.49–2.40	2.69–2.60
Unique Reflections (last shell)	14,344 (1311)	10,601 (929)
Completeness (%) (last shell)	98.6 (90.3)	92.8 (80.3)
Average I/σ_I_	15.4	8.8
R_merge_^b^ (%)	9.0	9.4
**Crystallographic refinement**		
Resolution range (Å)	28.9–2.40	35.3–2.60
Reflections	14,338	10,595
Rms deviation from ideality		
Bond lengths (Å)	0.005	0.005
Bond angles (°)	0.75	0.76
Rotamer outliers	0.0	0.0
Ramachandran		
Outliers	0.0	0.0
Allowed	100%	100%
Favored	98.7%	97.8%
R value (%)	19.1	19.0
R_free_ (%)	23.9	24.6

INPP1, inositol polyphosphate 1-phosphatase; PDB, Protein Data Bank.

a Resolution limit was defined as the highest resolution shell, where the average I/σ_I_ was 2.

b R_merge_ = ∑_hkl_∑_i_∣I_i(hkl)_ − <I_(hkl)_>∣/∑_hkl_∑_I_I_(hkl)_.

### Lithium inhibition of the INPP1-PP1-D54A mutant

Li^+^ inhibition of the D54A mutant INPP1 was determined as follows: 25 μl reactions containing 2.4 μg of enzyme, 40 μM (4 × K_m_) Ins(1,3,4)P_3_, ∼2000 cpm of ^3^H-Ins(1,3,4)P_3_, 50 mM HEPES, pH 7.5, 1 mM EGTA, 3 mM MgCl_2_, and 0 to 100 mM LiCl (to a total of 100 mM with KCl, to keep ionic strength constant) were incubated at 37 °C for 15 min, then diluted with 0.5 ml of 0.35 M NH4COO, 0.01 M COOH (IP_3_ formate) to stop the reactions. Stopped reaction mixtures were divided into two fractions and passed over 200 μl AG 1-X8 (200–400 mesh, formate form) anion exchange columns (Bio-Rad). The columns were each washed with 3 ml IP_3_ formate and the eluate (containing only Ins(3,4)P_2_ product) collected into scintillation vials containing 12 ml of Tru-Count high salt capacity scintillation fluid (Tru-Lab). Vials were counted in a Packard 1600 TR liquid scintillation analyzer. Percent hydrolysis of the substrate was 20 to 40% for each of the reactions. Nonradioactive Ins(1,3,4)P_3_ was purchased from Matreya, and ^3^H-Ins(1,3,4)P_3_ was prepared by digesting 1 μCi of ^3^H-Ins(1,3,4,5)P_4_ with ∼100 ng of type I 5-phosphatase for 1 h at 37 °C, stopping the reaction at 100 °C for 10 min, then centrifuging the mixture to clear the 5-phosphatase. An aliquot of the supernatant was run on an anion exchange HPLC column to confirm complete digestion of the substrate. The V_max_ and K_m_ values for the D54A INPP1 mutant were determined by similar kinetic studies using 0 to 50 μM of Ins(1,3,4)P_3_ in the absence of Li^+^.

All kinetic data were evaluated using the program Prism (GraphPad Software, Inc). V_max_ and K_m_ values for the D54A mutant were determined by fitting kinetic data by nonlinear regression to the one site binding hyperbola equation Y = V_max_∗X/(K_d_ + X) with an r^2^ value = 0.99 and the kinetic parameters determined. Data for Li^+^ inhibition for the D54A mutant and wild-type enzyme were fit by linear regression with r^2^ values of 0.95 and 0.99, respectively, to a plot of 1/v *versus* [Li^+^], for the hydrolysis of Ins-1,3,4-P_3_. The K_i_ value was determined according to the following equation: X int = −K_i_ (1 + K_M_/[Ins-1,3,4-P_3_]). All enzyme activities are the averages of three independent measurements.

## Data availability

Most data are provided in the manuscript; however, diffraction data and coordinates have been deposited in the Protein Data Bank for following INPP1 complexes: INPP1/Mg^2+^/Li^+^ (1INP), INPP1/Gd^3+^ (6WRO), INPP1/Gd^3+^/Li^+^ (6WRR), INPP1/Gd^3+^/Ins(1,3,4)P_3_ (6WRY), and INPP1/Gd^3+^/Li^+^/Ins(1,3,4)P_3_ (6X25); INPP1^D54A^/Ca^2+^ (7KIO); INPP1^D54A^/Ca^2+^/IP_2_ (7KIR)

## Conflict of interest

The authors declare that they have no conflicts of interest with the contents of this article.
